# Evaluating the Association Between Depressive Symptoms and Glycemic Control Among Residents of Rural Appalachia

**DOI:** 10.13023/jah.0403.03

**Published:** 2023-01-01

**Authors:** Brittany L. Smalls, Adebola Adegboyega, Ellen Combs, Eli W. Travis, Felipe De La Barra, Lovoria B. Williams, Nancy Schoenberg

**Affiliations:** University of Kentucky College of Medicine, brittany.smalls@uky.edu; University of Kentucky; University of Kentucky; University of Kentucky College of Medicine

**Keywords:** Appalachia, depression, diabetes, self-care, rural health

## Abstract

**Introduction:**

Type 2 diabetes mellitus (T2DM) is associated with a range of co-morbid physical and psychological conditions, including depression. Yet there is a dearth of evidence regarding the prevalence of depression among those in Appalachia living with T2DM; this gap persists despite the higher regional prevalence of T2DM and challenging social determinants of health.

**Purpose:**

This study aimed to provide greater detail about the relationships between T2DM and depressive symptoms in adults living in Appalachia Kentucky.

**Methods:**

The present study was a cross-sectional analysis of baseline data derived from an ongoing study of Appalachia Kentucky adults living with T2DM. Outcome data included demographics, Center for Epidemiologic Studies Depression Scale, point-of-care HbA1c, and the Summary of Diabetes Self-Care Activities. Bivariate analysis was conducted using Pearson’s correlation to determine the statistically significant relationships between variables which were then included in a multiple regression model.

**Results:**

The sample (N=365), consisted primarily of women (n=230, 64.6%) of mean age 64 years (±10.6); almost all (98%) were non-Hispanic White (n=349), and most were married (n=208, 59.1%). The majority (47.2%) reported having two comorbid conditions (n=161), including T2DM, and the mean HbA1c was 7.7% (1.7). Nearly 90% were nonsmokers (n=319). Depressive symptoms were reported in 25% (n=90) of participants. A higher number of comorbid conditions, increased age, Medicaid insurance, tobacco use, lower financial status, female sex, and disability compared to fully employed status all were correlated with a higher rate of depressive symptoms (r ≤ 0.2). The regression indicated that depressive symptoms were associated with age (β = −0.010, *p* = 0.001); full-time employment status compared to those who are disabled (β = −.0209, *p* = 0.18); men compared to women (β = −0.122, *p* = 0.042), and those who smoke compared to nonsmokers (β = 0.175, *p* = 0.038).

**Implications:**

Depressive symptoms were correlated with T2DM among this sample of Appalachian residents with poorly controlled T2DM, especially among women. Given the vast number of social determinants (e.g., poverty, food insecurity, and rurality) affecting this population, healthcare providers must assess for depression and consider its negative influence on the patient’s ability to achieve glycemic control.

## INTRODUCTION

Diabetes is a health crisis that affects approximately 34.2 million U.S. adults and causes distress within rural communities.^1^ Type 2 diabetes mellitus (T2DM) is associated with a multitude of factors, including obesity, environmental factors, sedentary lifestyle, and cigarette smoking.^2^,^3^ The Centers for Disease Control and Prevention (CDC) produced a map of a geographically distinct area in the southern U.S. known as the “Diabetes Belt,” where the adult T2DM prevalence is 11%—much higher than the national average of 8.5%.^2^ One-third of Diabetes Belt counties are located in central and southern Appalachia,^2^ and studies have found that residents in rural and economically distressed Appalachian counties had a 33% higher risk [95% confidence interval (CI), 1.10–1.60] of reporting diabetes than residents of non-Appalachian counties.^3^ These statistics show the need to address diabetes disparities across Appalachia, where the effects are remarkable.

In addition to higher rates of T2DM, rural dwellers exhibit higher prevalence of comorbidities—such as obesity, hypertension, and hyperlipidemia—than their urban counterparts.^4^,^5^ They are also challenged by interrelated social determinants of health, such as poor access to health care and food insecurity. These conditions interplay in a continuum that leads to health disparities and further complicates efforts to address T2DM and its social and cultural underpinnings.^3^,^6^–^8^

Depression is one of the major comorbidities associated with T2DM. Clinically significant depression is present in one out of every four people with T2DM^9^, and it is one of the leading causes of mental illness in the U.S., second only to anxiety.^10^ Depression rates across the Appalachian Medicare population are markedly increased, at a rate of 16.7% compared to the national average of 15.4%.^11^ In rural Appalachia, those living with T2DM are two times more likely to also experience depression.^14^

Depressive symptoms can diminish ability for self-care, subsequently leading to suboptimal health outcomes.^12^,^13^ Among those who simultaneously live with T2DM, depression can lead to high blood glucose, functional disability, medical noncompliance, and increased mortality.^9^,^14^ An existing diagnosis of depression has been associated with the likelihood for developing T2DM as well as disruption to disease management.^12^ Patients with depression report decreased compliance with treatment regimens, as well as feelings of distress and frustration.^15^,^16^ Furthermore, stress associated with depressive symptoms can increase cortisol levels, which hamper regulation of glucose and can contribute to hypertension and insulin resistance.^16^

Adherence to T2DM self-care activities can be challenging for residents living in geographically isolated communities, even when comorbidities are not present. For instance, Appalachia is impacted by barriers stemming from low socioeconomic status and an isolated geographic location marked by decreased resources and limited access to medical care.^17^ The link between food access and healthy eating illustrates these challenges. According to the U.S. Department of Agriculture, in 2015, 12.3% of Kentucky residents lived in low-income and low-access (LILA) census tracts, with more than 1 mile (urban areas) or more than 10 miles (rural areas) from the nearest supermarket, supercenter, or large grocery store.^18^ Several counties in Appalachia have been designated rural food deserts—low-income census tracts with a poverty rate ≥ 20%, where ≥ 33% of residents reside more than 10 miles from a large grocery store—by the U.S. Department of Agriculture.^19^ Owing to the high proportion of food deserts in Appalachia, many of the region’s residents do not have access to grocery stores and instead purchase food items from convenience stores that are rarely stocked with healthy choices.^3^ Food insecurity makes it difficult to follow healthy eating advice for T2DM and is associated with inadequate self-care of T2DM.^18^ Other regional challenges, such as poverty, contribute to difficulty adhering to treatment regiments. Poverty, in particular, is a known predictor of depression^15^ and may alter mental behavior leading to unhealthy habits, such as healthy eating, substance use, and reduced physical activity.^16^,^20^

These alarming and abounding factors must be addressed to improve well-being among Appalachian residents. Still, the interplay of T2DM, depression, and selfcare has not been well observed in a community-based sample of Appalachian adults. Given that residents of Appalachia have higher rates of T2DM, depression, and a 27% increased risk of comorbid T2DM and depression,^21^ the present study examined and describes the relationships between depression, glycemic control, and T2DM self-management practices among Appalachian residents. By understanding this relationship, healthcare professionals and researchers will be able to provide care and develop interventions that will be most effective in rural communities.

## METHODS

### Study Overview

This paper reports the results of baseline cross-sectional data collected as part of the ongoing study “Community to Clinic Navigation to Improve Diabetes Outcomes” (R01 DK112136, PI: Schoenberg). The baseline data collection included a diverse array of behaviors (e.g., self-care behaviors) and psychosocial factors (e.g., depression) relevant to glycemic control. Study approval was obtained by the Office of Research Integrity at the University of Kentucky.

### Recruitment

Participants were recruited through community sites within six counties in rural Appalachia Kentucky, classified by the Appalachian Regional Commission with a Rural–Urban Commuting Area (RUCA) code of 10, indicating a rural, geographically isolated area that is economically distressed, with high rates of unemployment, poverty, and low income.^22^,^23^ Counties were selected because of their resource constraints and health profiles, and owing to the research group’s longstanding engagement with local organizations within them. The mountainous, 54-county Appalachian region of Kentucky comprises a significant portion of rural Central Appalachia and contains the majority of economically distressed areas within the larger Appalachian Region.^24^

Community recruitment offers advantages over clinical recruitment, including enrolling hard-to-reach individuals with impeded access to clinics, avoiding selection bias of healthier participants with better clinic access, increasing comfort and trust of participants, and ensuring that the facilities are accessible to participants after hours.^25^ Participants were eligible to participate if they were 18 years or older, lived in Appalachia Kentucky with no plans to relocate out of the area within the next 18 months, showed a willingness and ability to participate (i.e., no major cognitive impairment), and had a diagnosis of T2DM and/or hemoglobin A1c (HbA1c) levels ≥ 6.5%. Individuals interested and potentially eligible were asked to complete written consent forms and undergo point-of-care HbA1c to confirm eligibility.

### Measures

#### Independent Variables

The Center for Epidemiologic Studies Depression (CES-D) Scale^26^ was used to measure depressive symptoms. The CES-D scale includes 20 items that assess aspects of depression, such as depressed mood, feelings of guilt and worthlessness, helplessness and hopelessness, and sleep disturbance. Responses use a 4-point Likert scale: 0 (rarely, less than 1 day), 1 (some of the time, 1 to 2 days), 2 (a moderate amount of time, 3 to 4 days), or 3 (most or all of the time, 5 to 7 days). Scores range from 0 to 60, with higher scores indicating increased severity of depression. Scores of 16 or higher are considered indicative of depression.^27^

#### Outcome Variables

Outcomes of interest for this study included glycemic control and T2DM selfcare practices. Glycemic control was measured using point-of-care HbA1c values. The Summary of Diabetes Self-Care Activities (SDSCA) was used to assess key self-care components, including general diet; specific diet (e.g., fruit/vegetable and fat consumption); exercise; blood sugar self-testing; and taking medication.^28^ The subscale scores were then averaged to create an overall measure of self-care.^28^

#### Demographic Variables

The following variables were included as covariates: age, sex, race/ethnicity, marital status, education level, employment status, insurance status, and financial status. The number of chronic conditions were determined using the following question: “Has a doctor, nurse, or other health professional ever told you that you had any of the following?” The responses included heart attack, coronary heart disease/angina, stroke, kidney disease, high blood pressure, high cholesterol, T2DM, type 1 diabetes, and Hepatitis C.^29^

### Statistical Analysis

Descriptive statistics using means and standard deviations were used to display the distribution of each variable included in the analysis. Bivariate analysis then was conducted using Pearson’s correlation to determine the statistically significant relationships between variables. Next, a multiple regression model controlling for relevant covariates was conducted. Statistical significance was determined at *p* < 0.05. All data analysis was conducted using Stata version 14.

## RESULTS

Table 1 displays a description of the sample (N=356). The sample consisted primarily of women (n=230, 64.6%), mean age 64 years (± 10.6); almost all (98%) were non-Hispanic White (n=349), and most were married (n=208, 59.1%). Forty-four percent (n=154) indicated that they had *just enough to get by* as their financial status. Education level was almost the same for high school (n=115, 32.3%) and graduate degree (n=113, 31.7%). This was a highly educated sample; about one-third of the participants in this study had at least a bachelor’s degree, but data from Appalachia Kentucky shows that 24.9% of adults ages 25 and over have at least a bachelor’s degree.^22^ Employment status varied among the group; however, the collective majority were disabled (n=73, 20.5%), employed full-time (n=63, 17.7%), or retired (n=150, 42.1%). Most participants reported having two chronic conditions (n=161, 47.2%), including T2DM which was poorly controlled, and a mean HbA1c of 7.7% (±1.7). The majority (89.6%) were nonsmokers (n=319). Overall, nearly one-fourth of the sample (n=90) reported experiencing depressive symptoms.

The relationship between depressive symptoms and personal factors is shown in Table 2. Using Pearson’s correlation, there were statistically significant correlations (*p* < 0.05) between depressive symptoms and the number of health conditions (r = 0.123), age (r = −0.21), Medicaid insurance (r = 0.2), smoking status (r = 0.185), sex (r = −0.16), employment status (r = −0.11), and financial status (r = 0.15). Though these correlations were statistically significant (*p* ≤ 0.5), the strength of these relationships were relatively week (r ≤ 0.2).

Linear regression was used to further analyze the relationship between depressive symptoms, controlling for personal factors (see Table 3). The regression indicated that depressive symptoms were not significantly associated with HbA1c or self-care activities. However, there were statistically significant negative associations between depressive symptoms and age (β = −0.010, *p* = 0.001); full-time employment status compared to those who are disabled (β = −0.209, *p* = 0.18); and men compared to women (β = −0.122, *p* = 0.042). Contrarily, there was a positive statistically significant association between depressive symptoms and those who smoke compared to nonsmokers (β = 0.175, *p* = 0.038). Similar to the results of the Pearson’s correlation, the β coefficients of the regression are statistically significant, but relatively weak. Interestingly, for both the correlation and regression analysis, self-care was shown not to have a statistically significant relationship with depressive symptoms in this sample population.

## DISCUSSION

The results of this study add to the existing literature by exploring the intersection of T2DM, rural Appalachia, and depressive symptoms. The study examines a culturally unique people group in geographical area with an exceptionally high prevalence of T2DM. Few studies exist that analyze the relationship between glycemic control and depressive symptoms in rural Appalachia residents living with T2DM.^14^ Previous studies have shown that those living with T2DM are 41% more likely to show signs of depression than those not diagnosed with T2DM.^30^ It is important to understand the impact of depressive symptoms in this geographically isolated population, especially since there is a known association with poorer physical and mental efficacy, as well as poorer compliance to diabetes self-care recommendations.^31^ In the study by Ciechanowski et al.,^31^ 26.7% of participants reported depressive symptoms, which is higher than the rate of depression reported nationally (15.4%)^24^; yet, this rates is similar to the rate of 27% reported in another study conducted with patients with T2DM in rural Appalachia.^32^

The findings of this study are consistent with those of others. For instance, prior studies have established the relationship of comorbid conditions and the higher prevalence of depression among older adults.^33^ Previous studies have also shown that there is a positive correlation between the number of chronic health conditions and depression.^34^ However, little data exist on how or if that correlation holds true in the unique region of Appalachia, where there is a high burden on residents with poor health status.^35^ The results of this study provide insight into the relationship between depressive symptoms and glycemic control in this vulnerable and underserved population. As such, these finding stress the importance of physician awareness and screening for depression in T2DM patients with an increased number of health conditions among the population studied.^33^ Given that the study population consisted of exclusively patients with T2DM, it could be expected that an increased correlation between age and depression would be found. Results confirm that this correlation holds true within the population of those living with T2DM in rural Appalachia. These findings underscore the importance of established care and increased depression screening among older adults.

Women in the general population are disproportionally affected by depression; thus, our findings are consistent with this knowledge.^36^ These findings among the vulnerable Appalachian population have important implications for healthcare providers. A 2012 study found that nearly 57% of women experiencing depression went undiagnosed.^37^ To assess for depression in the general population, the U.S. Preventive Services Task Force (USPSTF) issued a Grade B recommendation for the implementation of adequate systems to screen, diagnose, and follow up depression during every patient encounter.^38^ In rural Appalachia, healthcare providers and others who treat women must increase their awareness of the high prevalence and negative effects of depression among this population and adopt the USPSTF guideline for depression screening. There is little evidence to support a specific screening method, therefore healthcare providers can select the validated tool of their choice. Some tools, such as the brief, two-question PHQ-2 are easily administered during a time-constrained patient encounter.^39^

Previous studies have linked financial hardship, unemployment, and depressive symptoms.^40^ Our results indicate that higher rates of depressive symptoms were shown in those with perceived financial trouble as well as those receiving disability benefits compared to those who are employed full time. The close relationship between financial status and employment status suggests a comprehensive approach to these two personal factors. In patients with T2DM in rural Appalachia, requiring an annual depression screening for those receiving disability benefits should be considered. A questionnaire assessing financial status and employment status should be administered and used to determine which patients are at highest risk for developing depressive symptoms.

Literature shows that poverty and smoking status are strongly correlated,^41^ but the correlation between smoking and depression is not a clearly established link based on the current published literature.^42^ In this study of rural Appalachia, however, the data show a positive correlation between smoking status and depressive symptoms. Regardless of whether smoking causes depression (or vice versa), this finding supports providing increased depression screening among patients who smoke. Offering concurrent depression screening with smoking cessation counseling could be supported by this finding. A link between smoking and depression provides another supportive argument to prioritize smoking cessation counseling.

There was no significant relationship between depression, glycemic control, and self-care practices in this study. This is not in consonance with previous literature that have shown that depression is associated with poor glycemic control and self care^12^,^43^,^44^ among patients with diabetes. This may be a function of the overall valuable asset of social support that may be available for rural Appalachian residents and serve as buffer against stressors.^45^ The lack of association between depression, glycemic control, and self-care practices among patients with T2DM has implications for research and practice. It shows the need for holistic management of each of these factors. The rural healthcare providers are positioned to provide proper screening, management, and monitoring for depression to improve patient outcomes. In addition, researchers should test interventions that include self-care education and support to promote self-care and glycemic control among patients with T2DM.

While the results of this study offer key insights into the relationship between T2DM and depression in Appalachia, there are limitations. First, the correlations found between personal factors and depression status was relatively weak, ranging from an absolute r coefficient of 0.11 to 0.21. Second, the sample size of the study was only 356 participants. Larger studies conducted in the future may produce more informative results. Third, diabetes distress is more common among patients with T2DM and clinically depressed individuals, but it was not measured in this study. Future studies would benefit from inclusion of a diabetes distress scale. Lastly, the study enrolled participants on a voluntary basis. This has the potential to create volunteer bias, in that the participants may be more likely to comply with treatment than those who did not volunteer. In addition, participants were enrolled from community centers and churches; these participants are probably healthier, and more active than those who are depressed and more likely to isolate themselves from activities.

Further exploration in this area of study could involve larger sample sizes to produce a more robust data analysis. Additional studies are needed to assess efficacy of interventions in T2DM patients with depression, including how the interventions are affected by the personal factor variables measured in this study. Finally, future studies should be developed that leverage the existing community assets, such as social support and community connectedness, to develop interventions for this population.

**SUMMARY BOX**

**What is already known about this topic?**

Previous studies have linked financial hardship, unemployment, and depressive symptoms. However, literature on the relationship between these factors, T2DM, and self-care is lacking, especially when it comes to community-based studies in Appalachia.

**What is added by this report?**

This study explores the intersection of T2DM, self-care, and depressive symptoms among a rural Appalachian population. Its results provide insight into the relationship between depressive symptoms and glycemic control in this vulnerable and underserved population. Positive correlations were found between age and depression and being impoverished and depression. Furthermore, the data showed a positive correlation between smoking status and depressive symptoms. There was no significant relationship between depression, glycemic control, and self-care practices.

**What are the implications for future research?**

Future studies should use a larger sample size to confirm the results seen here. Additional studies are needed to assess efficacy of interventions in T2DM patients with depression, including how the interventions are affected by the personal variables measured in this study. Finally, future studies should be developed that leverage the existing community assets, such as social support and community connectedness, to develop interventions for this population.

## Figures and Tables

**Table 1:** Study sample characteristics (N=356)

Variables	Mean (SD) or N (%)	Missing
**Age**	64 (±10.6)	3

**Gender**		0
*Male*	126 (35.4)	
*Female*	230 (64.6)	

**Race**		0
*Non-Hispanic White*	349 (98.0)	
*Other*	7 (1.9)	

**Financial status**		8
*Just about enough to get by*	154 (44.2)	
*More than you need to live well*	91 (26.1)	
*Sometimes struggle to make ends meet*	103 (29.6)	

**Employment**		0
*Disabled*	73 (20.5)	
*Full-time*	63 (17.7)	
*Homemaker*	49 (13.7)	
*Part-time*	12 (3.3)	
*Retired*	150 (42.1)	
*Unemployed*	9 (2.5)	

**Education**		0
*High school*	115 (32.3)	
*Associates degree*	43(12.1)	
*Bachelor’s*	24 (6.7)	
*Graduate degree*	113 (31.7)	
*Some college*	61 (17.1)	

**Marital status**		4
*Divorced*	55 (15.6)	
*Married*	208 (59.1)	
*Never married*	21 (5.9)	
*Widowed*	68 (19.3)	

**Insurance status**		0
*Uninsured*	7 (2.0)	
*Insured*	349 (98.0)	

**Number of health conditions in addition to T2DM**		15
0	17 (4.9)	
1	72 (21.1)	
2	161 (47.2)	
3	58 (17.0)	
4	24 (7.0)	
5	7 (2.1)	
6	2 (0.5)	

**Self-care composite score**	17.1 (±6.3)	1

**Smoking status**		0
*Yes*	37 (10.4)	
*No*	319 (89.6)	

**Depressive symptoms**		19
*Yes*	90 (26.7)	
*No*	247 (73.2)	
**HbA1c**	7.7 (±1.7)	28

**Table 2:** Pearson’s correlation of depressive symptoms and personal factors (n=337)

Variables	Depressive Symptoms *r coefficient*
HbA1c	0.09
Self-Care	−0.13
Number of Health Conditions	0.123*
Age	−0.21*
Insurance	−0.02
Medicaid	0.20*
Race/Ethnicity	0.041
Employment Status	−0.11*
Sex	−0.16*
Education Status	0.012
Financial Status	0.150*
Marital Status	−0.030
Smoking Status	0.185*

NOTES:

* *p* < 0.05.

**Table 3:** Adjusted regression model of the association between depressive symptoms, self-care, and HbA1c (n=337)

Variables	Depressive Symptoms	
	*β coefficient*	P-*value*
HbA1c	0.010	0.522

Self-Care	−0.006	0.125

Number of Health Conditions	0.041	0.089

Age	−0.010	0.001*

Medicaid		
*No*†	--	--
*Yes*	0.051	0.454

Race/ethnicity		
*Other*†	--	--
*Non-Hispanic White*	0.074	0.729

Employment Status		
*Disabled*†		
*Fulltime*	−0.209	0.018*
*Homemaker*	−0.045	0.633
*Part time*	0.189	0.164
*Retired*	−0.049	0.505
*Unemployed*	0.047	0.769

Sex		
*Female*†	--	--
*Male*	−0.122	0.042*

Education Status		
*High school*†	--	--
*Associates degree*	−0.006	0.938
*Bachelor’s*	−0.083	0.456
*Graduate degree*	−0.034	0.587
*Some college*	0.035	0.646

Financial Status		
*Just about enough to get by*†	--	--
*More than you need to live well*	−0.086	0.172
*Sometimes struggle to make ends meet*	0.024	0.712

Marital Status		
*Divorced*†	--	--
*Married*	−0.077	0.328
*Never married*	−0.242	0.062
*Widowed*	−0.038	0.676

Smoking Status		
*No*†		
*Yes*	0.175	0.038*

NOTES: This model was adjusted for the following sociodemographic characteristics: Medicaid, number of health conditions, age, race/ethnicity, education, sex, financial status, marital status, and smoking status.

* *p* < 0.05

† Reference group for the regression model.
